# e-INEBRIA Special Interest Group Roadmap for Best Practices for Research on Brief Digital Interventions for Problematic Alcohol and Illicit Drug Use

**DOI:** 10.2196/20368

**Published:** 2020-08-14

**Authors:** Michael Patrick Schaub, Anne H Berman, Hugo López Pelayo, Nikolaos Boumparis, Zarnie Khadjesari, Matthijs Blankers, Antoni Gual, Heleen Riper, Lodewijk Pas

**Affiliations:** 1 Swiss Research Institute for Public Health and Addiction ISGF University of Zurich Zurich Switzerland; 2 Centre for Psychiatry Research Department of Clinical Neuroscience Karolinska Institutet Stockholm Sweden; 3 Stockholm Health Care Services Stockholm County Council Stockholm Sweden; 4 Department of Psychology Uppsala University Uppsala Sweden; 5 Grup Recerca Addicions Clínic (GRAC-GRE) Department of Psychiatry, Clinical Institute of Neuroscience Hospital Clínic i Universitari de Barcelona, Universitat de Barcelona Barcelona Spain; 6 Institut d'Investigacions Biomèdiques August Pi i Sunyer (IDIBAPS) Barcelona Spain; 7 La Red de Trastornos Adictivos, RETICS Madrid Spain; 8 Department of Clinical, Neuro- and Developmental Psychology Amsterdam Public Health Research Institute Vrije Universiteit Amsterdam Amsterdam Netherlands; 9 School of Health Sciences University of East Anglia Norwich United Kingdom; 10 Trimbos Institute The Netherlands Institute of Mental Health and Addiction Utrecht Netherlands; 11 Department of Research Arkin Mental Health Care Amsterdam Netherlands; 12 Amsterdam UMC, Location AMC Department of Psychiatry University of Amsterdam Amsterdam Netherlands; 13 Hospital Clinic Psychiatry Department Neurosciences Institute Barcelona Spain; 14 Department of Clinical, Neuro- and Developmental Psychology Faculty of Behavioural and Movement Sciences Vrije Universiteit Amsterdam Amsterdam Netherlands; 15 Academic Center General Practice Catholic University Leuven Leuven Belgium

**Keywords:** brief interventions, mobile applications, good practice, implementation research, quality assurance

## Abstract

There is great potential for scaling up the delivery of brief interventions for alcohol and illicit drug use, given the increasing coverage of mobile devices and technologies for digital interventions, including apps for smartphones and tablets. However, while the number of digital interventions is increasing rapidly, the involvement of brief-intervention researchers and the development of good practices has just begun. In 2018, the Special Interest Group on digital interventions of the International Network on Brief Interventions for Alcohol & Other Drugs (e-INEBRIA SIG) initiated a conversation regarding possible avenues of future research, which subsequently became a roadmap for digital interventions. This roadmap consists of points considered relevant for future research, ongoing technological developments, and their implementation across a continuum of prevention and care. Moreover, it outlines starting points for the diversification of brief digital interventions, as well as next steps for quality improvement and implementation in public health and clinical practice. The roadmap of the e-INEBRIA SIG on digital interventions is a starting point that indicates relevant next steps and provides orientation for researchers and interested practitioners with regard to the ambiguous literature and the complexity of current digital interventions.

## Background

The Special Interest Group on digital interventions of the International Network on Brief Interventions for Alcohol & Other Drugs (e-INEBRIA SIG) was launched at the INEBRIA conference in Lausanne (2016); the group also took part in INEBRIA conferences in 2017 (New York) and 2018 (Santiago de Chile). During the conference in Santiago de Chile, e-INEBRIA SIG discussed possible avenues for further research that subsequently, in continued meetings, turned into a roadmap, as presented in this commentary. In particular, e-INEBRIA SIG is interested in the potential of electronic health (eHealth) technologies for brief interventions in health and other settings to reduce the harms produced by alcohol, drug use, and gambling.

There is great potential for scaling up the delivery of brief interventions, given the increasing coverage in high, middle, and low income countries of smartphones, wearables, and other connected devices worldwide, and the rise of new technologies like geotagging and chatbots. Knowing which technologies can be successfully applied in target groups of alcohol and drug users and which components of interventions are essential for reducing harm could help enhance their impact at a public health level.

One strategy to foster evidence-based developments is to keep track of ongoing scientific developments, including periodic reviews and meta-analyses on the effectiveness and implementation of internet interventions for alcohol and other substances. Recent meta-analyses have demonstrated the effectiveness of internet interventions at reducing alcohol use in alcohol misusers [1]; reducing cannabis use in cannabis misusers, at least in the short-term [2]; and reducing drug use among opioid and mixed-drug users [3]. Moreover, individual patient data (IPD) meta-analyses could, due to their large pooled samples, help to identify initial trends in subgroups (eg, [1]).

However, a limitation of most digital interventions developed as part of scientific studies is that they often lack funding for maintenance after study completion. A recent review on such interventions in the alcohol field identified 72 trials assessing alcohol interventions, among which only 8 (11%) remained accessible; these were mostly internet-based interventions [4].

While numerous digital interventions aiming to reduce substance use are available, most lack evidence-based brief intervention content, have never been evaluated for effectiveness, and have been developed without academic or specialist input, including some that are profit-oriented [5,6]. In the case of cannabis misuse, the situation is worse because the few genuine apps aimed at harm reduction and reducing cannabis use are forced to compete with hundreds of currently available apps that promote cannabis use [7]. Some attempts have been made to develop evidence-based health app quality rating scales, and one has been developed for researchers [8], with a version available for end users [9]; however, it is unlikely that alcohol and drug users make use of the latter. Thus, it is rational to foster research on genuine harm-reduction–oriented digital interventions, particularly apps, to promote their use and evidence-based quality development.

While it is rational to develop generalizable digital interventions to reduce harm from alcohol and drug use at a general population level, there is evidence from other mental health fields that tailored digital interventions for specific cultures or migrants [10] can increase their effectiveness. There are few systematic reviews of tailored digital interventions for specific alcohol- or drug-using groups and classical diversification topics (eg, different age groups, like the elderly, as well as different genders, educational levels, and cultures). The development of such digital interventions in the addiction field is still in its infancy relative to face-to-face interventions.

## Roadmap for Brief Digital Interventions

### Aims

Taking into consideration the complexity of current brief digital interventions, the aim of the roadmap is to propose further steps and provide orientation in the ambiguous literature for researchers and interested practitioners thus promoting the coordinated, systematic development of a knowledge base that will facilitate the effective development and application of digital interventions.

### Methods

The members of e-INEBRIA SIG represent 6 European countries, have previously developed and evaluated digital interventions for alcohol and other drugs, and exchange information and plan further research at the annual INEBRIA conference. We developed a preliminary roadmap during the last INEBRIA preconference workshop in 2018, in Santiago, Chile, and refined it during subsequent bimonthly teleconferences and email exchanges. The roadmap was then organized into four main topic groups and, once again, discussed within the group.

### Topics

#### Evaluation of Effective Implementation Modalities

- Continue evaluation research efforts (start to construct an ongoing collection of overall data sets for IPD meta-analyses; begin with meta-analyses for different interventions and target groups)
- Investigate which elements make digital interventions more effective, particularly to identify components that improve adherence, enhance motivation, and/or promote sustained behavior change
- Investigate how brief digital interventions should be integrated and can enhance different forms of blended or face-to-face treatments for alcohol and drug use disorders (with continuous electronic monitoring or personalized feedback; investigate the potentials and limitations of motivational interviewing)
- Investigate the complementarity between anonymous applications for public health and applications for the clinical setting, including hazardous or harmful use recognition and identification; behavior change support; and continued harm reduction
- Investigate public health and clinical applications for specific vulnerable populations (eg, gender, age, cultural issues, personal differences, differences in contexts and settings).
- Develop a core set of validated outcome measures suitable for use in digital brief intervention research, depending on the targeted substance

#### Diversification and Cultural Sensitivity

- Develop and investigate easy-to-use and tablet-optimized guided and unguided brief interventions for the elderly, with extended content and adapted measures
- Foster the development of culturally generalizable interventions for newly industrialized countries, in collaboration with the World Health Organization (WHO) and other relevant international organizations
- Start to adapt brief digital interventions for specific cultures and minorities to investigate if cultural adaptation makes a difference in an intervention’s acceptance and effectiveness

#### Accommodation of New Technology

- Encourage research on novel technologies, such as chatbots, for brief internet interventions to allow for more interactivity, including geotagging functions (eg, to warn against places that are risky for relapses or to network with supporting peers or social workers in the immediate proximity at concert events [5])
- Clarify whether and how brief digital interventions can profit from gamification and for which target groups
- Develop and investigate just-in-time adaptive brief digital interventions
- Make use of big data technologies (eg, as an additional data source for missing data within a classical randomized controlled trial; to derive a natural additional control group in addition to existing groups in a classical randomized controlled trial design; or to gain better access to risk groups for brief digital interventions at population levels).
- Develop an evidence base of effective implementation strategies, where training, support, and financial incentives are the most effective strategies for implementing face-to-face brief interventions

#### Intervention Quality and Safety Management

- Develop quality criteria for digital interventions to change alcohol use and illicit drug use and promote harm reduction
- Develop a directive on collaboration between research and commercial interests, particularly for publicly funded organizations and private collaborations
- Further develop the taxonomy for describing brief digital interventions in a standardized way
- Develop best practices for professionals working with brief digital interventions
- Keep abreast of ongoing quality developments, like genuine health app rating websites

## Discussion

While this roadmap provides orientation to current developments in this field, it is also considered a starting point. It will need updating and ongoing promotion at future meetings and conferences, as well as in publications. Moreover, we must ensure that the points listed in our roadmap will be followed by actions. Therefore, we plan to continue our discussions between future INEBRIA conferences, during symposia and workshops at these conferences, and at related conferences like those of the International Society for Research on Internet Interventions (ISRII) and the International Society of Behavioral Medicine (ISBM), where alcohol and drug use can be linked to relevant developments in digital interventions (ISRII), epidemiology, and interventions for health-related behaviors and chronic conditions (ISBM).

## Conclusions

The roadmap proposed by e-INEBRIA SIG on digital interventions is a starting point that indicates relevant next steps. It also provides orientation for researchers and interested practitioners with regard to the ambiguous literature and the complexity of current digital interventions. Moreover, it is a call for action to coordinate our efforts toward research and evidence-based implementation in the context of rapid technological development.

## Abbreviations

eHealth: electronic health
e-INEBRIA SIG: Special Interest Group on digital interventions of the International Network on Brief Interventions for Alcohol & Other Drugs (INEBRIA)
IPD: individual patient data
ISBM: International Society of Behavioral Medicine
ISRII: International Society for Research on Internet Interventions
WHO: World Health Organization
